# Determinants of suboptimal immune recovery among a Chinese Yi ethnicity population with sustained HIV suppression

**DOI:** 10.1186/s12879-022-07113-y

**Published:** 2022-02-08

**Authors:** Liyu Chen, Chang-Hai Liu, Shuang Kang, Lingyao Du, Fanghua Ma, Changmin Li, Lang Bai, Hong Li, Hong Tang

**Affiliations:** 1 Center of Infectious Diseases, West China Hospital of Sichuan University, No.37 Guoxue Alley, Chengdu, 610041 Sichuan China; 2 Division of Infectious Diseases, State Key Laboratory of Biotherapy and Center of Infectious Diseases, West China Hospital, Sichuan University, Chengdu, China; 3 Center of Antiretroviral Treatment, People’s Hospital of Zhaojue County, 616150 Liangshan, Yi Autonomous Prefecture China

**Keywords:** HIV-infected Yi ethnicity population, Sustained HIV suppression, Immune recovery

## Abstract

**Objectives:**

Despite sustained viral suppression with effective antiretroviral therapy (ART), HIV-infected patients with suboptimal immune recovery are still at high risk of both non-AIDS-related and AIDS-related events. The aim of this study was to investigate determinants potentially associated with suboptimal CD4 + T cell count recovery during free ART with sustained viral suppression among an HIV-infected Yi ethnicity population in Liangshan Prefecture, an area in China with high HIV prevalence.

**Methods:**

This retrospective study included HIV-infected Yi adults (≥ 18 years and baseline CD4 + T cell count less than 500 cells/μL) for whom ART supported by National Free Antiretroviral Treatment Program was initiated between January 2015 and December 2018 in Zhaojue County, Liangshan Prefecture. Virological suppression (viral load < 50 copies/mL) was achieved within 12 months after ART initiation, and sustained virological suppression was maintained. Multivariate log-binomial regression analysis was used to assess determinants of suboptimal immune recovery.

**Results:**

There were 140 female and 137 male patients in this study, with a mean age of 36.57 ± 7.63 years. Most of the Yi patients were infected through IDU (48.7%) or heterosexual contact (49.8%), and the anti-HCV antibody prevalence was high (43.7%, 121/277). Of the 277 patients with a mean ART duration of 3.77 ± 1.21 years, complete immune recovery occurred in only 32.9%. The baseline CD4 + T cell count in patients with suboptimal and intermediate immune recovery was 248.64 ± 108.10 and 288.59 ± 108.86 cells/μL, respectively, which was much lower than the baseline 320.02 ± 123.65 cells/μL in patients who achieved complete immune recovery (*p* < 0.001). Multivariable analysis demonstrated that low pre-ART CD4 + cell count and coinfection with HCV were associated with immune recovery of the HIV patients.

**Conclusions:**

Our study suggests that for HIV-infected Yi patients in Liangshan Prefecture, prompt ART initiation after diagnosis of HIV infection should be applied, and curative HCV treatment should be given to patients with HCV/HIV coinfection to improve the immunological effectiveness of ART.

*Trial registration* None

**Supplementary Information:**

The online version contains supplementary material available at 10.1186/s12879-022-07113-y.

## Strength and limitations of this study

1. This was a retrospective study including 277 HIV-infected Yi ethnicity patients with sustained viral suppression in Liangshan Prefecture (the area with the highest HIV prevalence in China) to investigate potential determinants associated with suboptimal CD4 + T cell count recovery.
2. All included patients were on NNRTI- or protease inhibitor-based ART regimens, precluding assessment of immune recovery in patients on integrase-based ART regimens.
3. HCV RNA levels were not measured in this study; therefore, patients with anti-HCV antibody positivity but low HCV RNA viral load may have been included.

## Introduction

Since its first report in 1981, HIV/AIDS has remained one of the world’s most serious public health challenges [1]. According to the United Nations Program on HIV/AIDS (UNAIDS), 38.0 million people (36.2 million adults, 1.8 million children) globally lived with HIV/AIDS in 2019. Of these, 1.7 million were newly infected in 2019 (https://www.unaids.org/en). In China, the number of people living with HIV surpassed 1 million by the end of 2020, with more than 12,000 people being newly infected (http://www.nhc.gov.cn/wjw/). Although the overall national prevalence of HIV/AIDS in China remains low, prevalence varies greatly in different regions and among different ethnic populations.

In Liangshan Yi Autonomous Prefecture, which is located in Sichuan Province, Southwest China, and is a high epidemic area, 53.84% of the population is of Yi ethnicity (approximately 2.86 million). Currently, all six counties (Butuo, Zhaojue, Meigu, Yuexi, Jinyang and Puge) in China with HIV/AIDS prevalence surpassing 1% are located within Liangshan Prefecture. Most of the HIV-infected individuals in Liangshan Prefecture are Yi, and the dominant transmission routes are injection drug use (IDU) and heterosexual contact [2–5]. The high prevalence of HIV infection among the Yi population can be partly ascribed to the geographical location of Liangshan Prefecture and the culture of the Yi people. On the border with Yunnan Province, Liangshan Prefecture has long been an important channel for drug smuggling from the Golden Triangle into Sichuan Province, and the use of heroin has even been socially acceptable among the Yi people [2, 3, 6]. Poverty, casual sex, including extramarital sex and concurrent sexual partnerships, are also risk factors for the high HIV prevalence in this region.

To effectively control the HIV/AIDS epidemic in Liangshan Prefecture, China’s National Free Antiretroviral Treatment Program (NFATP) [7–9] provides free anti-retroviral therapy (ART) to all people living with HIV in the area. It should be noted that integrase inhibitors have not been included in NFATP, and thus most of the ART regimens provided are non-nucleoside reverse transcriptase inhibitor (NNRTI) or protease inhibitor based. Through widespread implementation, the percentage of patients with access to ART continues to increase, enabling an growing number of HIV/AIDS patients to achieve and maintain an undetectable viral load. As undetectable equals untransmissible [10, 11], NFATP in Liangshan Prefecture has made a great contribution to the effective control of HIV transmission. Nevertheless, we observed that a considerable proportion of HIV-infected Yi patients fail to show CD4 + T cell recovery to a normal level (> 500 cells/μL), despite achieving and maintaining sustained HIV suppression, and these patients with suboptimal immune recovery are still at high risk of non-AIDS-related and AIDS-related events. To further improve ART efficacy, the aim of this study was to investigate potential determinants associated with suboptimal CD4 + count recovery in HIV-infected Yi individuals who have achieved sustained viral suppression under China’s NFATP in Zhaojue County, Liangshan Prefecture.

## Materials and methods

### Study design and patients

**Fig. 1:**
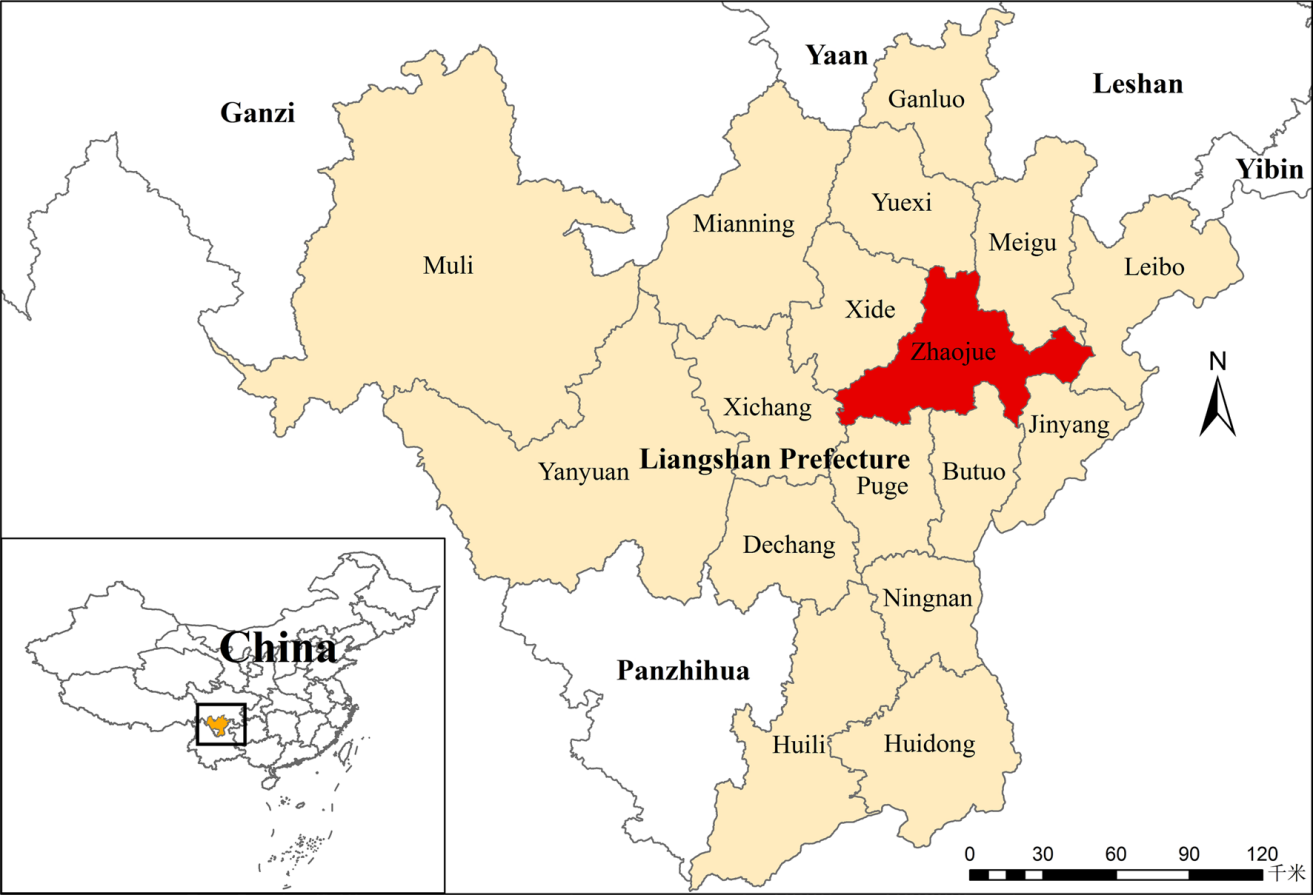
Map of Liangshan Yi Autonomous Prefecture in Sichuan Province, Southwest China. All HIV-infected Yi adults included in this study were from Zhaojue County (labelled red)

This retrospective study included all HIV-infected Yi adults (≥ 18 years and baseline CD4 + T cell count less than 500 cells/μL) who initiated ART supported by NFATP between January 2015 and December 2018 in Zhaojue County, Liangshan Prefecture (Fig. 1), achieving virological suppression (viral load < 50 copies/mL) within 12 months after ART initiation and maintaining sustained virological suppression. Based on a recent study [12], immune recovery was stratified by CD4 + T cell count measured at the most recent follow-up visit, as follows: complete immune recovery (CD4 ≥ 500 cells/μL), intermediate immune recovery (350 ≤ CD4 < 500 cells/μL), and suboptimal immune recovery (CD4 < 350 cells/μL). All HIV-infected Yi patients in this study were from Zhaojue County of Liangshan Prefecture. As integrase inhibitors have not been included in NFATP, the current first-line NFATP ART regimen consists of NNRTI, either efavirenz (EFV) or nevirapine (NVP), or a protease inhibitor, either lopinavir/ritonavir (LPV/r) in combination with tenofovir disoproxil fumarate (TDF)/lamivudine (3TC) or zidovudine (AZT)/3TC.

CD4 + T cell count was measured by flow cytometry (BD-FACSCalibur), and HIV RNA in plasma was evaluated using the Roche Cobas TaqMan HIV-1 (HPS) test every 12 months until the end of 2020. Thus, the longest follow-up time was 72 months, and the shortest follow-up time was 24 months. Anti-hepatitis C virus (HCV) antibodies and hepatitis B surface antigen (HBsAg) were measured by ELISA with HCV antibody and HBsAg test kits, respectively (KHB, China). In addition, other routine follow-up tests, including haematology, urinalysis and blood liver function, were performed every 6 months; renal function for patients taking TDF was assessed every 6 months, as was the serum lipid level for patients taking LPV/r.

Anaemia was defined as haemoglobin less than 120 g/L for men and 110 g/L for women. Liver function test abnormalities were defined as alanine aminotransferase (ALT) > 40 U/L, aspartate aminotransferase (AST) > 40 U/L or total bilirubin (TBil) > 17.1 µmol/L. Based on a recent study, the CD4/CD8 ratio was categorized into three groups: < 0·30, 0·30–0·45, and > 0·45 (> 0.45 as reference) [13]. The body mass index (BMI) of the subjects was calculated by dividing the subject’s weight (kg) by the square of their height (m^2^).

This study was approved by the Medical Ethics Committee of West China Hospital of Sichuan University (Annual Audit No. 450, Version 2020.5). The study was performed by following the ethical guidelines expressed in the Declaration of Helsinki and the International Conference on Harmonization Guidelines for Good Clinical Practice. Informed consent was obtained from all subjects.

### Statistics

Data were extracted from NFATP Data System. Data were reported as the mean ± standard deviation for normal continuous variables and median (interquartile range) for non-normal continuous variables; frequency was used for discrete variables. Multivariate log-binomial regression models were used to assess determinants of immune recovery, with adjusted odds ratios (aORs) and confidence intervals (CIs). Confounders or predictors for each outcome were analysed as covariates according to their biological plausibility. The nonstatistical expert-based selection method was used to determine variables to include in the multivariate regression model [14, 15]. Variance inflation factor (VIF) and tolerance (1/VIF) values were employed to determine collinearity between factors included in multivariable analysis. All *p* values were 2-sided, and *p* < 0.05 was considered statistically significant. Statistical analyses were performed using the statistical software SPSS (Version 24.0, IBM, Armonk, New York, USA).

## Results

**Fig. 2:**
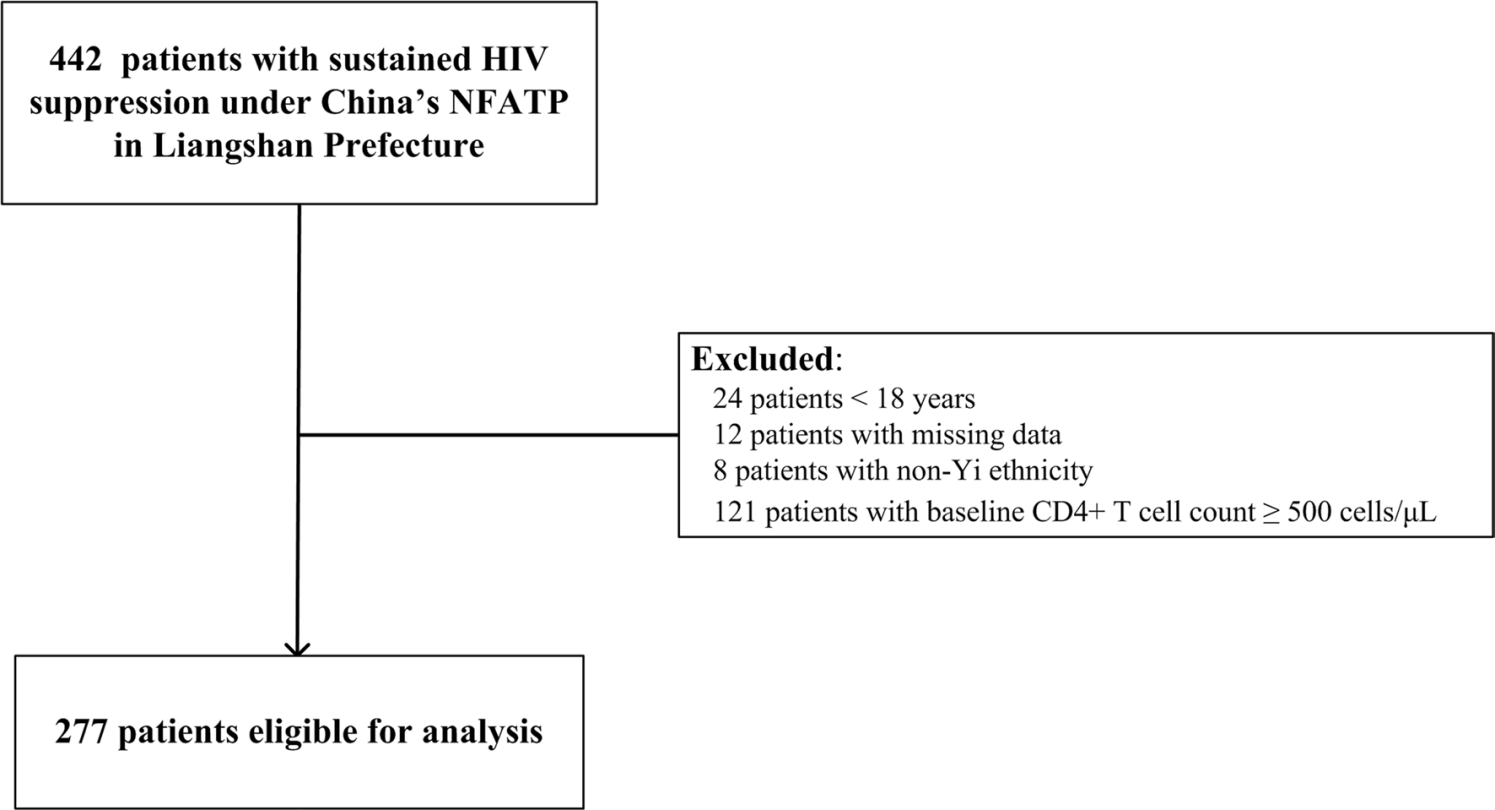
Flow chart of this study

Between January 2015 and December 2018, 277 HIV-infected Yi patients (with baseline CD4 + T cell count less than 500 cells/μL) who initiated ART and achieved sustained virological suppression were included in this study to investigate the incidence of suboptimal immune recovery and associated determinants (Fig. 2). There were 140 female and 137 male patients in this study, with a mean age of 36.57 ± 7.63 years. Most of the Yi patients were infected through IDU (48.7%) or heterosexual contact (49.8%).

The baseline CD4 + T cell count in patients with suboptimal and intermediate immune recovery was 248.64 ± 108.10 and 288.59 ± 108.86 cells/μL, respectively, which was much lower than the baseline 320.02 ± 123.65 cells/μL in patients who achieved complete immune recovery (*p* < 0.001). In contrast, the prevalence of anti-HCV antibody positivity in patients with suboptimal and intermediate immune recovery was much higher than that in patients with complete immune recovery (55.0% vs. 39.5% vs. 35.2%, *p* < 0.001) (Table 1).

**Table 1:** Patients’ characteristics at ART initiation

Variables	Suboptimal immune recovery group (n = 100)	Intermediate immune recovery group (n = 86)	Complete immune recovery group (n = 91)	*p* value
Sex				0.037
Female (%)	55 (55.0%)	47 (54.7%)	35 (38.5%)	
Male (%)	45 (45.0%)	39 (45.3)	56 (61.5%)	
Age (years)	38.01 ± 7.70	36.19 ± 8.02	35.34 ± 7.00	0.462
CD4 + T cell count (cells/μL)	248.64 ± 108.10	288.59 ± 108.86	320.02 ± 123.65	< 0.001
CD4/CD8 ratio	0.31 ± 0.23	0.34 ± 0.21	0.37 ± 0.22	0.179
HIV viral load (log_10_ copies/mL)	5.23 ± 4.86	5.20 ± 4.85	5.21 ± 4.86	0.527
BMI (kg/m^2^)				0.061
< 18.5	19 (19.0%)	5 (5.8%)	9 (9.9%)	
18–24.5	75 (75.0%)	73 (84.9%)	77 (84.6)	
> 24.5	6 (6.0%)	8 (9.3%)	5 (5.5%)	
HBsAg positivity (%)	9 (9.0%)	11 (12.8%)	13 (14.3%)	0.507
Anti-HCV positivity (%)	55 (55.0%)	34 (39.5%)	32 (35.2%)	0.014
WHO stage (%)				0.495
I and II	96 (96.0%)	85 (98.8%)	88 (96.7%)	
III and IV	4 (4.0%)	1 (1.2%)	3 (3.3%)	
ART regimens (%)				0.423
EFV + TDF + 3TC	62 (62.0%)	59 (68.6%)	58 (63.7%)	
EFV + AZT + 3TC	30 (30.0%)	18 (20.9%)	25 (27.5%)	
NVP + AZT + 3TC	5 (5.0%)	2 (2.3%)	4 (4.4%)	
NVP + TDF + 3TC	1 (1.0%)	2 (2.3%)	0 (0.0%)	
LPV/r + AZT + 3TC	1 (1.0%)	4 (4.7%)	4 (4.7%)	
LPV/r + TDF + 3TC	1 (1.0%)	1 (1.2%)	0 (0.0%)	
Treatment duration (years)	3.93 ± 1.19	3.58 ± 1.25	3.79 ± 1.21	0.155
Leucocyte	6.49 ± 3.73	6.51 ± 2.08	6.50 ± 2.10	0.999
Platelet	170.13 ± 71.57	178.33 ± 62.78	181.10 ± 69.96	0.513
Hemoglobin (g/dL)	139.11 ± 23.53	161.20 ± 219.73	138.84 ± 22.77	0.387
TBil	13.34 ± 9.67	13.85 ± 8.22	15.06 ± 9.70	0.424
ALT	40.16 ± 38.55	37.76 ± 31.76	40.78 ± 47.20	0.867
AST	40.38 ± 33.47	45.84 ± 54.67	40.58 ± 35.39	0.614

AZT, zidovudine; 3TC, lamivudine; LPV/r, Lopinavir/ ritonavir; EFV, efavirenz; NVP, nevirapine; TDF, tenofovir disoproxil fumarate; TBil, total bilirubin; ALT, alanine aminotransferase; AST, aspartate aminotransferase

The prevalence of HBsAg positivity among the patients with suboptimal, intermediate, and complete immune recovery was 9.0%, 12.8%, and 14.3%, respectively (*p* = 0.507). Most of the included Yi patients were in WHO clinical stages I and II (269/277, 97.1%), only 8 patients (2.9%) were in stages III and IV. The ART regimens were mostly based on EFV (91%, 251/277), followed by NVP (5.1%, 14/277) and LPV/r (3.9%, 11/277) in combination with TDF/3TC or AZT/3TC. Other variables, including the baseline CD4/CD8 ratio, HIV viral load, BMI, treatment duration, blood cell count, haemoglobin, and liver function, were comparable among the three groups of patients with different immune recoveries (Table 1).

**Fig. 3:**
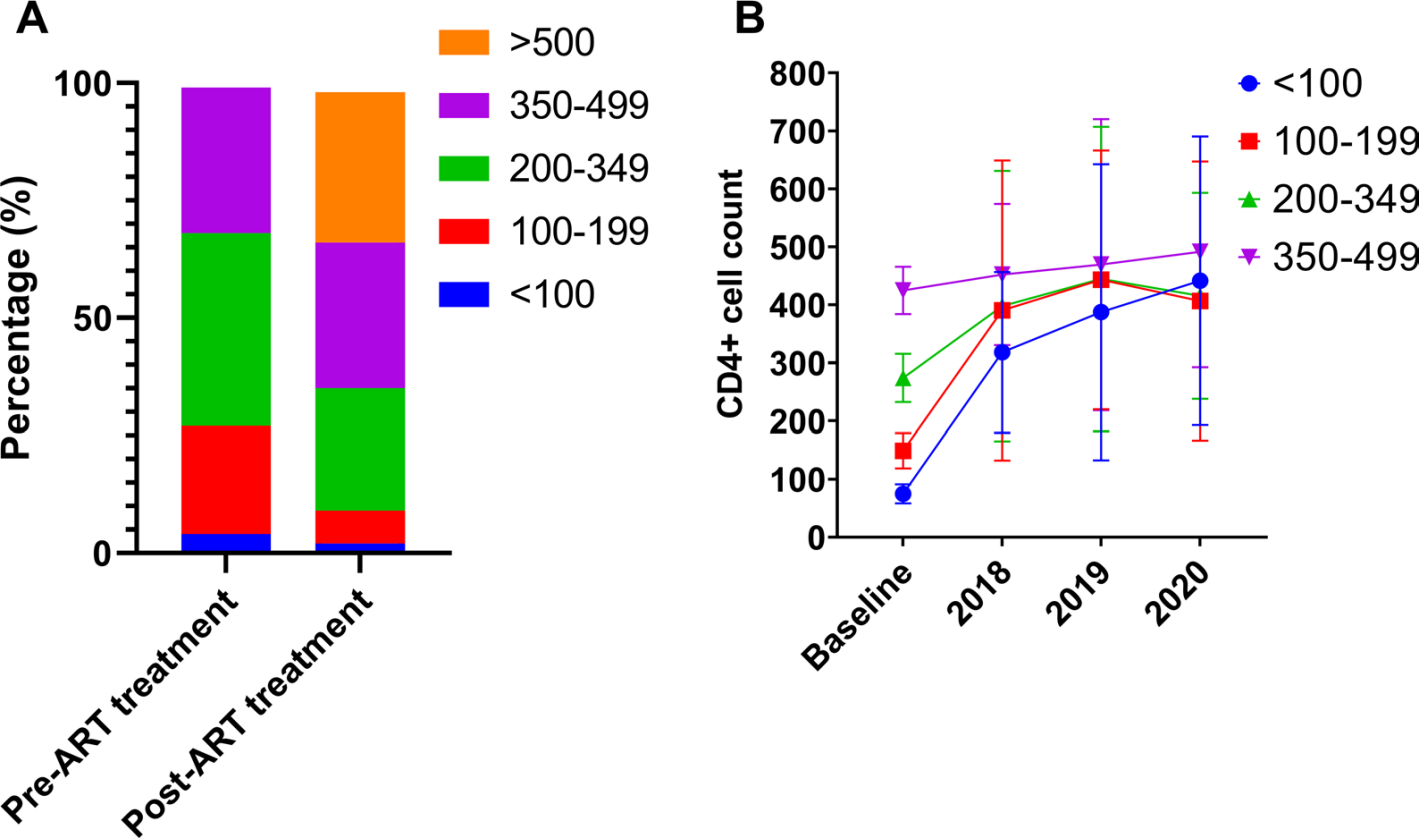
The change of CD4 + T cell count with ART treatment. **A** Proportion of Yi patients (n = 277) stratified by CD4 + T cell count at baseline and the most recent follow-up visit post-ART; **B** The trends of CD4 + T cell count during the follow-up

During the follow-up period, the proportion of patients with CD4 + T cell count ≥ 350 cells/μL increased from 31.4% (87/277) to 63.9% (177/277), and the proportion of patients with CD4 + T cell count ≥ 200 cells/μL increased from 72.9% (202/277) to 90.3% (250/277) (Fig. 3A). However, of the 277 patients with a mean ART duration of 3.77 ± 1.21 years of ART, complete immune recovery occurred in only 32.9% of the included patients (Fig. 3B).

Multivariable analysis was carried out to further investigate potential determinants associated with suboptimal CD4 + count recovery in this population. A lower baseline CD4 + cell count was found to significantly influence achieving CD4 + cell recovery > 500 cells/mL. Specifically, compared with patients with a pre-ART CD4 + cell count ≥ 350 cells/μL, those with a pre-ART CD4 + cell count in the range of 200–350 cells/μL (aOR 0.422, 95% CI 0.212–0.839, *p* = 0.014), 100–199 cells/μL (aOR 0.421, 95% CI 0.871–0.895, *p* = 0.022), and < 100 cells/μL (aOR 0.478, 95% CI 0.245–0.874, *p* = 0.017) had a lower chance of achieving a CD4 + cell count > 500 cells/μL (Table 2). However, a lower baseline CD4 + cell count was not found to significantly influence achieving CD4 cell recovery > 200 and > 350 cells/mL. Of note, the number of patients with a baseline CD4 + cell count < 100 cells/mL was very small (11/277). Interestingly, coinfection with HCV was associated with a lower chance of achieving a CD4 + T cell count > 200 cells/μL (aOR 0.215, 95% CI 0.073–0.633, *p* = 0.005), > 350 cells/μL (aOR 0.45, 95% CI 0.245–0.834, *p* = 0.011), and > 500 cells/μL (aOR 0.261, 95% CI 0.095–0.716, *p* = 0.009). Taken together, we demonstrate that a low pre-ART CD4 + cell count and coinfection with HCV are associated with immune recovery in HIV patients.

**Table 2:** Determinants associated with suboptimal CD4 + count recovery among Yi patients with sustained HIV suppression on ART

	CD4 + cell count > 200 cells/mL		CD4 + cell count > 350 cells/mL		CD4 + cell count > 500 cells/mL	
	aOR (95% CI)	*p* value	aOR (95% CI)	*p* value	aOR (95% CI)	*p* value
Age category (years)						
18–29	Reference		Reference		Reference	
30–39	1.186 (0.289–4.871)	0.812	0.698 (0.327–1.489)	0.352	1.001 (0.497–2.016)	0.998
40–49	1.257 (0.280–5.650)	0.765	0.684 (0.293–1.596)	0.380	0.694 (0.307–1.569)	0.380
≥ 50	1.081 (0.080–14.645)	0.954	0.462 (0.119–1.793)	0.265	0.847 (0.203–3.530)	0.820
Male sex	2.272 (0.791–6.524)	0.127	1.258 (0.681–2.321)	0.464	1.908 (1.017–3.580)	0.044
Pre-ART CD4 + count						
< 100	0.710 (0.101–4.991)	0.730	1.140 (0.285–4.559)	0.853	0.478 (0.245–0.874)	0.017
100–199	Reference		0.707 (0.356–1.402)	0.321	0.421(0.871–0.895)	0.022
200–350			Reference		0.422 (0.212–0.839)	0.014
350–500					Reference	
Pre-ART CD4/CD8 ratio						
> 0.45	Reference		Reference		Reference	
0.30–0.45	0.062 (0.006–0.603)	0.017	0.824 (0.347–1.959)	0.661	0.635 (0.289–1.398)	0.260
< 0.30	0.164 (0.017–1.624)	0.122	0.704 (0.306–1.617)	0.408	0.655 (0.302–1.422)	0.284
HIV viral load (copies/ml)						
< 100 000	Reference		Reference		Reference	
> 100 000	0.746 (0.229–2.434)	0.628	0.604 (0.295–1.236)	0.168	0.935 (0.473–1.848)	0.846
BMI (kg/m^2^)						
< 18.5	0.844 (0.242–2.943)	0.790	0.399 (0.173–0.920)	0.031	0.749 (0.300–1.871)	0.536
18.5–24	Reference		Reference		Reference	
> 28	0.814 (0.263–2.367)	0.998	1.359 (0.448–4.123)	0.588	0.822 (0.257–2.625)	0.740
HBsAg positive	9.098 (0.964–85.897)	0.054	1.445 (0.584–3.577)	0.426	1.299 (0.569–2.965)	0.534
Anti-HCV positive	0.215 (0.073–0.633)	0.005	0.453 (0.245–0.834)	0.011	0.261 (0.095–0.716)	0.009
WHO clinical stage						
I and II	Reference		Reference		Reference	
III and IV	0.104 (0.010–1.045)	0.054	0.541 (0.108–2.703)	0.454	1.197 (0.227–6.303)	0.832
Treatment duration > 3 years	0.555 (0.192–1.602)	0.277	0.552 (0.304–1.001)	0.050	0.900 (0.502–1.614)	0.725
Leucocyte < 4 or > 10 × 10^9^/L	1.408 (0.318–6.243)	0.653	1.295 (0.552–3.034)	0.552	1.451 (0.621–3.389)	0.389
Platelet < 100 × 10^9^/L	2.125 (0.433–10.432)	0.353	0.589 (0.249–1.393)	0.228	0.892 (0.347–2.296)	0.813
Anemia	0.445 (0.104–1.906)	0.275	0.836 (0.341–2.049)	0.695	0.856 (0.327–2.243)	0.752
TBil > 17.1 µmol/L	0.979 (0.333–2.883)	0.970	1.663 (0.841–3.289)	0.144	1.824 (0.945–3.520)	0.073
ALT > 40 U/L	0.914 (0.255–3.273)	0.890	1.159 (0.525–2.560)	0.715	1.136 (0.512–2.524)	0.753
AST > 40 U/L	0.617 (0.179–2.127)	0.444	1.225 (0.570–2.629)	0.603	1.084 (0.503–2.337)	0.837

WHO clinical stage was not associated with immune recovery, which might be ascribed to the very small number of patients in stages III and IV (8/277, 2.9%). Other variables, including age, male sex, baseline CD4/CD8 ratio, viral load, BMI, HBsAg, treatment duration, blood cell count, anaemia and liver function, were also not associated with immune recovery in this study.

To avoid the unexpected bias from different ART regimens, we excluded the 26 patients on non-EFV-based regimen and only included the 251 patients on EFV-based ART in a newly generated multivariable analysis. Similarly, low pre-ART CD4 + cell count and coinfection with HCV were found to be associated with suboptimal immune recovery in this population (Additional file 1: Table S1).

## Discussion

With the advent of ART, HIV infection has been transformed from a death sentence to a chronic but treatable disease for most infected individuals. Nonetheless, approximately 30% of patients receiving ART fail to exhibit CD4 + T cell recovery to a normal level, despite achieving sustained HIV suppression [12, 16, 17]. Persistently low CD4 + T cell counts are associated with an increased risk of both AIDS-related and non-AIDS-related morbidity and mortality [18–20]. Thus, identification of all possible risk factors involved in poor CD4 + T cell recovery may provide clinicians guidance in choosing the most effective treatment approaches for these patients [17]. In the present study of 277 HIV-infected Chinese Yi ethnicity patients evaluated after a mean ART of 3.77 ± 1.21 years, we observed that a low baseline CD4 + cell count and HIV/HCV coinfection are associated with immune recovery in HIV patients.

To our knowledge, this is the first study to analyse risk factors potentially associated with suboptimal immune recovery in HIV-infected Yi ethnicity patients in Liangshan Prefecture, Southwest China. Through multivariable logistic regression analysis, we found a low pre-ART CD4 + cell count to be an independent risk factor for incomplete immune recovery in HIV patients, which is consistent with previous studies [12, 17, 21–23]. It has been reported that many individuals who start ART at CD4 + cell count < 350 cells/μL never achieve a normal CD4 + cell count ≥ 500 cells/μL, even after up to 10 years of effective ART [22, 24]. Thus, the current international guidelines recommending that prompt or even immediate ART initiation on the day of HIV positivity diagnosis is of great importance to individuals living with HIV before they have markedly decreased CD4 + T cell counts and progression to AIDS [25, 26].

In the present study, the prevalence of HBV/HIV coinfection was 11.9%, which was comparable to the HBV/HIV coinfection rate (13.85%) recently reported among HIV-infected patients in Guangxi Zhuang Autonomous Region [27]. The HBV infection rate among HIV-infected individuals is much higher than that of the general population (5–6%) in China [28]. Consistent with previous studies [28–31], our study revealed that HBV status did not influence the CD4 + T cell response after ART initiation (Table 2). However, it needs to be emphasized that progression of chronic HBV infection to severe end-stage liver disease, including liver failure, cirrhosis, and hepatocellular carcinoma, occurs more rapidly in individuals with HBV/HIV coinfection than in individuals with HBV mono-infection [18, 32]. Thus, in patients with HBV/HIV coinfection, TDF or TAF in combination with FTC should be the backbone of ART because these three drugs are active against both viruses [18, 25, 33]. The ART backbone used in this study was mostly TDF/3TC (66.4%), with the remaining 33.6% being AZT/3TC (Table 1). It is expected that the ART backbone AZT/3TC will be gradually removed from China NFATP and replaced by TDF/emtricitabine (FTC).

The prevalence of anti-HCV antibody positivity among the 277 HIV-infected Yi patients included in this study was 43.7%, which may be explained by the fact that 48.7% of the patients were infected through IDU. Both HCV and HIV are blood-borne viruses, and the presence of HIV infection increases the transmission efficiency of HCV [34]. It has been reported that HCV prevalence in HIV-infected individuals is much higher than that in HIV-negative individuals, especially in people who inject drugs [34]. HIV coinfection accelerates HCV progression, as patients with HCV/HIV coinfection have a 2.92-fold greater risk of experiencing progression to severe liver diseases than do patients with HCV mono-infection [35]. However, the effect of HCV infection on CD4 + T cell recovery after efficient ART remains controversial. Some studies reported that the CD4 + T cell recovery was comparable between HCV/HIV coinfected patients and HIV-mono-infected patients on efficient ART [36] or that clearance of HCV replication did not influence immune recovery [37]. In contrast, several studies have found that despite sustained HIV suppression due to efficient ART, HCV/HIV coinfected patients have a lower probability of achieving optimal immune recovery than patients with HIV mono-infection [38–42]. Our present study also revealed a negative correlation between HCV infection and optimal CD4 + T cell recovery (Table 2), suggesting that all patients with HCV/HIV coinfection should be evaluated for curative HCV treatment [18]. With the advent of direct-acting antiviral (DAA) therapies, HIV/HCV coinfection is no longer difficult to treat [43]. Of note, when concurrent treatments for both HIV and HCV are indicated, careful consideration needs to be taken regarding drug-drug interactions between ART drugs and HCV DAAs [44]. In the present study, more than 95% of the ART regimens were EFV- and NVP-based (Table 1). The DAA regimen sofosbuvir/ledipasvir is recommended for HCV eradication, whereas the DAA regimens sofosbuvir/velpatasvir, elbasvir/grazoprevir, and glecaprevir/pibrentasvir are contraindicated. As velpatasvir, elbasvir, grazoprevir, and glecaprevir are substrates of cytochrome P450 3A (CYP3A), their serum concentrations become reduced when co-administrated with EFV and NVP (CYP3A inducers) [18, 45].

Our study has limitations. First, all included patients were on NNRTI-based or protease inhibitor-based ART regimens, precluding assessment of immune recovery in patients on integrase-based ART regimens. Second, HCV RNA levels were not measured in this study; therefore, patients with anti-HCV antibody positivity but a low HCV RNA viral load may have been included. Third, there was a lack of data on smoking behaviour, which has been reported to play a role in suboptimal immune recovery [46, 47]. Fourth, the number of included Yi patients on efficient ART was relatively small, which may limit the identification of other determinants associated with suboptimal CD4 + T cell count recovery. Future studies are needed to include more Yi patients from Liangshan Prefecture to investigate more factors (e.g., cigarette smoking and alcohol consumption) likely to influence the immune recovery of HIV-infected patients on efficient ART [17, 21, 48, 49].

In summary, we demonstrate that a low pre-ART CD4 + cell count and HCV infection are associated with suboptimal CD4 + count recovery among HIV-infected Yi individuals on efficient ART in Liangshan Prefecture. Our study supports prompt ART initiation after HIV diagnosis and HCV eradication treatment in HCV/HIV coinfected patients by appropriate DAA regimens (compatible with HIV ART antiretrovirals), not only for HCV cure per se but also for improving the immunological effectiveness of ART.

## Supplementary Information

**Additional file 1: Table S1.** Determinants associated with suboptimal CD4 + count recovery among 251 Yi patients with sustained HIV suppression on EFV-based ART.

**Additional file 2.** Checklist for the appropriate reporting statement: STROBE.

## Data Availability

All data generated or analysed during this study are included in this published article and its additional files. Checklist for the appropriate reporting statement: STROBE (in Additional file 2).

## Abbreviations

UNAIDS: United Nations Program on HIV/AIDS
IDU: Injection drug use
ART: Anti-retroviral therapy
NFATP: National Free Antiretroviral Treatment Program
NNRTI: Non-nucleoside reverse transcriptase inhibitor
EFV: Efavirenz
NVP: Nevirapine
LPV/r: Lopinavir/ritonavir
TDF: Tenofovir disoproxil fumarate
3TC: Lamivudine
AZT: Zidovudine
HCV: Hepatitis C virus
HBV: Hepatitis B virus
TBil: Total bilirubin
ALT: Alanine aminotransferase
AST: Aspartate aminotransferase
aOR: Adjusted odds ratio
CI: Confidence interval
VIF: Variance inflation factor
CYP3A: Cytochrome P450-3A
