# Supplementary material for: Determinants of suboptimal immune recovery among a Chinese Yi ethnicity population with sustained HIV suppression
Source: BMC Infect Dis. 2022 Feb 8;22:137. doi: 10.1186/s12879-022-07113-y (PMC8827152; doi:10.1186/s12879-022-07113-y)
Supplement: Supplementary file 1 — Additional file 1: Table S1. Determinants associated with suboptimal CD4 + count recovery among 251 Yi patients with sustained HIV suppression on EFV-based ART. [file 12879_2022_7113_MOESM1_ESM.docx]

**Supplementary table 1. Determinants associated with suboptimal CD4+ count recovery among 251 Yi patients with sustained HIV suppression on EFV-based ART**

| Variables | CD4+ cell count >200 cells/ml | | CD4+ cell count >350 cells/ml | | CD4+ cell count >500 cells/ml | |
| --- | --- | --- | --- | --- | --- | --- |
|  | aOR (95% CI) | *p* value | aOR (95% CI) | *p* value | aOR (95% CI) | *p* value |
| Age category (years) |  |  |  |  |  |  |
| 18–29 | Reference |  | Reference |  | Reference |  |
| 30–39 | 0.721 (0.128-4.062) | 0.711 | 0.523 (0.223-1.226) | 0.136 | 0.687 (0.321-1.468) | 0.332 |
| 40–49 | 0.862 (0.138-5.368) | 0.874 | 0.533 (0.207-1.368) | 0.191 | 0.511 (0.213-1.227) | 0.133 |
| >=50 | 0.607 (0.035-10.457) | 0.731 | 0.353 (0.086-1.450) | 0.149 | 0.613 (0.143-2.631) | 0.510 |
| Male sex | 2.479 (0.702-8.750) | 0.158 | 1.160 (0.597-2.255) | 0.662 | 1.932 (0.989-3.773) | 0.054 |
| Pre-ART CD4+ count |  |  |  |  |  |  |
| <100 | 0.402 (0.048-3.348) | 0.399 | 0.733 (0.164-3.264) | 0.683 | 0.187 (0.049-0.765) | 0.014 |
| 100–199 | Reference |  | 0.712 (0.349-1.453) | 0.351 | 0.415 (0.175-0.982) | 0.045 |
| 200-350 |  |  | Reference |  | 0.321 (0.153-0.671) | 0.003 |
| 350-500 |  |  |  |  | Reference |  |
| Pre-ART CD4/CD8 ratio |  |  |  |  |  |  |
| >0.45 |  |  | Reference |  |  |  |
| 0.30-0.45 | 0.075 (0.007-0.792) | 0.031 | 0.938 (0.366-2.403) | 0.894 | 0.842 (0.360-1.969) | 0.692 |
| <0.30 | 0.179 (0.017-1.910) | 0.154 | 0.650 (0.267-1.582) | 0.343 | 0.799 (0.349-1.828) | 0.595 |
| HIV viral load (copies/ml) |  |  |  |  |  |  |
| <100 000 | Reference |  | Reference |  | Reference |  |
| >100 000 | 0.860 (0.247-2.987) | 0.812 | 0.508 (0.237-1.085) | 0.080 | 0.859 (0.424-1.739) | 0.673 |
| BMI (kg/m2) |  |  |  |  |  |  |
| <18.5 | 0.774 (0.208-2.879) | 0.702 | 0.513 (0.212-1.240) | 0.138 | 0.941 (0.362-2.449) | 0.901 |
| 18.5–24 | Reference |  | Reference |  | Reference |  |
| >28 | 0.835 (0.243-2.13) | 0.998 | 1.236 (0.383-3.986) | 0.722 | 1.053 (0.317-3.498) | 0.933 |
| HBsAg positive | 6.196 (0.610-62.899) | 0.123 | 1.120 (0.442-2.841) | 0.811 | 1.223 (0.513-2.919) | 0.650 |
| Anti-HCV positive | 0.199 (0.056-0.706) | 0.012 | 0.432 (0.221-0.842) | 0.014 | 0.284 (0.086-0.836) | 0.024 |
| WHO clinical stage |  |  |  |  |  |  |
| I and II | Reference |  | Reference |  | Reference |  |
| III and IV | 0.159 (0.010-2.516) | 0.192 | 0.808 (0.142-4.609) | 0.810 | 1.692 (0.294-9.718) | 0.556 |
| Treatment duration>3 years | 0.575 (0.183-1.800) | 0.342 | 0.561 (0.297-1.062) | 0.076 | 0.813 (0.437-1.513) | 0.513 |
| Leucocyte<4 or >10 ×10^9/L | 1.402 (0.275-7.153) | 0.685 | 1.402 (0.576-3.414) | 0.457 | 1.587 (0.645-3.906) | 0.315 |
| Platelet<100 ×10^9/L | 1.831 (0.361-9.274) | 0.465 | 0.555 (0.228-1.348) | 0.193 | 0.806 (0.300-2.165) | 0.669 |
| Anemia | 0.421 (0.087-2.037) | 0.282 | 0.776 (0.291-2.071) | 0.612 | 0.869 (0.302-2.503) | 0.795 |
| TBil>17.1 µmol/L | 0.779 (0.249-2.440) | 0.669 | 1.348 (0.661-2.746) | 0.411 | 1.504 (0.747-3.028) | 0.253 |
| ALT>40 U/L | 0.848 (0.223-3.231) | 0.809 | 0.967 (0.427-2.186) | 0.935 | 1.093 (0.473-2.525) | 0.835 |
| AST>40 U/L | 0.506 (0.135-1.894) | 0.311 | 1.352 (0.614-2.977) | 0.454 | 1.033 (0.464-2.299) | 0.937 |
